# The war on Gaza and its impact on public health: challenges and pathways to recovery

**DOI:** 10.3389/fpubh.2025.1664850

**Published:** 2025-10-13

**Authors:** Deema Al Bakri, Yousef Khader, Rana Khatib, Fawzi Al-Hammouri, Mousa Aabed, Yehia Abed, Yousef Zureikat, Omar Lattouf, Ahmad Shatat, Abdullatif Husseini, Mohannad Al Nsour, Haitham Bashier

**Affiliations:** ^1^The Eastern Mediterranean Public Health Network (EMPHNET), Amman, Jordan; ^2^Jordan University of Science and Technology, Irbid, Jordan; ^3^Birzeit University, Birzeit, Palestine; ^4^The Specialty Hospital, Amman, Jordan; ^5^Palestinian Ministry of Health, Nablus, Palestine; ^6^Juzoor Organization, Gaza, Palestine; ^7^Jordanian Royal Medical Services, Amman, Jordan; ^8^Emory University, Atlanta, GA, United States

**Keywords:** Gaza strip, public health emergency, health systems, humanitarian crisis, war-related health impacts

## Abstract

The ongoing humanitarian crisis in Gaza has created an unprecedented public health emergency, accompanied by the destruction of critical healthcare infrastructure, widespread shortages of essential resources, and the displacement of millions of the Gazan population. The escalation of conflict since October 2023 has severely strained Gaza’s already fragile health system. As a result, there has been an alarming increase in communicable diseases, malnutrition, and mental health challenges. Furthermore, healthcare facilities and healthcare workers have been systematically targeted, which has led the primary and emergency healthcare services to be on the brink of collapse and has led to significant gaps in immunization coverage. In response to these challenges, the Eastern Mediterranean Public Health Network (EMPHNET) organized two sessions during its Eighth Regional Conference in 2024. These sessions, “*Public Health in Gaza: Priorities and Solutions*” and “*The War on Gaza: Challenges and Opportunities for the Health Sector,”* gathered experts, policymakers, and healthcare professionals to discuss the immediate and long-term implications of the crisis and to call for action. Key areas of focus included emergency response planning, primary healthcare, communicable and non-communicable diseases, mental health support, and workforce capacity development. The discussions highlighted the urgent need for multi-sectoral collaboration and international support to address immediate healthcare needs and build a sustainable, resilient health system for Gaza. Recommendations included, first and foremost, ending the aggression, opening the borders, and stopping the illegal siege. Other recommendations included strengthening health governance structures, ensuring the protection of healthcare workers, and integrating innovative frameworks such as event- based disease surveillance and community-based mental health interventions. The sessions underscored the essential role of partnerships among local authorities, international organizations, and humanitarian agencies in fostering recovery and resilience. By prioritizing equity, resilience, and sustainability, the recommendations aim to guide efforts in rebuilding a functional and holistic healthcare system for Gaza’s future. The data and analysis presented reflect the status of Gaza’s public health system up to September 2024, based on insights shared during EMPHNET’s Eighth Regional Conference.

## Introduction

Over the past two decades, Gaza has faced a severe and ongoing public health crisis exacerbated by systemic challenges, including a complete blockade by air, water, and land since 2007 (1). This man-made crisis, compounded by significant damage to healthcare infrastructure and essential services, has left Gaza’s health system on the brink of collapse. Health challenges have been exacerbated by severe shortages of medications, medical supplies, equipment, laboratory testing, clean water, and food, as well as by the widespread displacement of the population (2).

The aggression that began in October 2023 caused large-scale destruction, leaving Gaza’s health system in a dire state. Hospitals, healthcare centers, and basic health services have been targeted, further crippling the ability to respond to the burgeoning public health demands (3). Compounding this, the emergence of communicable diseases, the strain on non-communicable disease management, and deteriorating mental health underline the high-priority need for comprehensive public health strategies (4, 5).

In light of these challenges, the Eastern Mediterranean Public Health Network (EMPHNET) organized two significant sessions during its Eighth Regional Conference in 2024. The *Gaza Forum: Public Health in Gaza: Priorities and Solutions* and the *Gaza Session: The War on Gaza, Challenges and Opportunities for the Health Sector* provided platforms for experts to discuss the immediate and long-term impacts of the war on Gaza’s health system. These sessions aimed to shed light on the public health priorities and challenges in Gaza and explore actionable strategies to address them. Bringing together experts, decision-makers, and other stakeholders, the sessions focused on highlighting the magnitude of the damage to Gaza’s public health system and proposing strategies for rebuilding and resilience.

The discussions in both the forum and the session emphasized the significant need for immediate and long-term interventions to address Gaza’s public health crisis. Specific focus areas included emergency response planning, primary healthcare, communicable and non-communicable diseases, mental health, and maternal and child health services. These areas were identified as essential for mitigating the immediate health impacts of the ongoing conflict and for building a more resilient healthcare system. Additionally, both sessions highlighted the importance of workforce capacity development, particularly in conflict settings, to ensure that healthcare providers are adequately trained to respond to emergencies.

This manuscript presents the outcomes and discussions held during the EMPHNET Eighth Regional Conference in September 2024. Therefore, the data and insights included herein reflect the status of the public health situation in Gaza up to that time. It is important to note that conditions have continued to deteriorate significantly since then, particularly throughout 2025, which falls beyond the scope of this paper.

## Sessions description

The *Gaza Forum* and *Gaza Session* were conducted in English and included presentations from a total of nine expert speakers, followed by interactive Q&A segments. The forum and session were moderated by Dr. Rana Khatib and Dr. Fawzi Al-Hammouri, respectively, who provided context on Gaza’s humanitarian situation and outlined the objectives. Dr. Khatib is the Vice President for Planning and Development at Birzeit University, Palestine, with extensive experience in public health, community health development, and humanitarian response. Dr. Al-Hammouri serves as the Executive Chairman of the Gaza Health Initiative and the Chairman of the Global Health and Relief Initiative, with a longstanding focus on improving healthcare. He is also the CEO of Specialty Hospital in Amman.

At the time of the conference in September 2024, these sessions offered timely insights into the public health emergency as it was unfolding and explored strategies needed to address both immediate and long-term health challenges. More than 380 individuals participated in the sessions, including representatives from international organizations, regional health authorities, humanitarian agencies, healthcare professionals, academics, and policymakers. Attendees included International Health Regulations (IHR) focal points, representatives from non-governmental organizations (NGOs), rapid response teams, and field epidemiology training program (FETP) graduates, as well as frontline healthcare providers.

The discussions featured insights from frontline healthcare providers, international organizations, and regional health authorities, providing a comprehensive exploration of both immediate needs and long-term resilience strategies for Gaza’s health system.

Dr. Mousa Aabed, Director General of Primary Health Care at the Palestinian Ministry of Health (MOH), provided a firsthand account of the current health crisis in Gaza. He highlighted the widespread damage to healthcare infrastructure, including attacks on health facilities and displacement of medical personnel, leading to severe shortages in medical supplies and fuel. Dr. Aabed detailed the collapse of Gaza’s immunization programs and the rise in communicable diseases, with over a million cases of respiratory infections reported since the start of the conflict. He emphasized the need for timely interventions to address displacement, supply shortages, and governance challenges.

Dr. Yehia Abed, a senior public health expert from Al-Quds University and the Director of Juzoor organization in the Gaza strip, discussed the dire humanitarian situation in northern Gaza, where health services have been severely disrupted. He shared firsthand experiences from medical points established in northern Gaza to provide critical healthcare amidst the destruction. Dr. Abed highlighted the psychological toll on healthcare workers and the population, emphasizing the urgent need for mental health services and support for managing non-communicable diseases.

Brigadier General Dr. Yousef Zureikat, Director General of the Royal Medical Services in Jordan, spoke about Jordan’s field hospitals operating in Gaza. He detailed the logistical and operational challenges of providing healthcare in a conflict zone, including supply chain issues and security risks. Dr. Zuraiqat emphasized the importance of effective emergency response planning and Jordan’s commitment to supporting Gaza’s healthcare sector through medical missions and field hospitals.

Professor Omar Lattouf, Professor of Surgery at Emory University, focused on the long- term impact of the conflict on Gaza’s healthcare infrastructure. He discussed the role of international collaboration in rebuilding healthcare systems and the importance of medical education and workforce capacity development. Dr. Lattouf stressed the need to strengthen partnerships and support local healthcare professionals to ensure sustainable recovery.

Dr. Ahmad Shatat, Acting Director General of International Cooperation at Gaza’s MOH, presented on the governance challenges in coordinating Gaza’s health recovery efforts. He highlighted the evolving governance structures aimed at facilitating recovery efforts and the difficulties in mobilizing international resources during the ongoing conflict. Dr. Shatat provided insight into local coordination efforts to address immediate and long-term healthcare needs.

Professor. Abdullatif Husseini, Professor of Public Health at the Institute of Community and Public Health, Birzeit University, discussed the broader public health implications of the ongoing war. He focused on the impact of the current war on managing non- communicable diseases (NCDs) and highlighted the efforts of the Gaza Health Initiative in capacity-building and education for healthcare professionals. Dr. Husseini underscored the need for tailored interventions to manage chronic diseases amidst the crisis.

Discussions across both sessions explored major areas for intervention, including:

- Emergency response planning
- Primary healthcare services
- Communicable and non-communicable diseases
- Mental health and psychosocial support
- Maternal and child health services
- Health governance and leadership
- Resource mobilization and international collaboration
- Workforce capacity development

These sessions underscored the importance of short-term relief efforts to address acute healthcare needs while promoting long-term strategies to rebuild a resilient and sustainable public health system in Gaza.

The discussions focused on key challenges and opportunities in rebuilding Gaza’s health sector, with specific attention to resource mobilization, governance reforms, and the integration of emergency preparedness into primary healthcare.

## Findings

### Destruction of Gaza’s healthcare infrastructure

Up to September 2024, the conflict in Gaza has caused catastrophic damage to the healthcare infrastructure where more than 1,000 attacks on health facilities since October 7, 2023, were reported. The majority of hospitals, clinics, and primary healthcare centers have been destroyed, severely compromising access to essential health services for Gaza’s population. The conflict has also led to the killing or displacement of healthcare workers, which has further put strain on the delivery of medical services due to decreased number of available healthcare workers. Panelists noted that the healthcare system in Gaza was already fragile due to years of blockade and siege and that the recent escalation had left it on the verge of collapse. The crisis has been worsened by shortages of medications, equipment, laboratory testing, fuel, food and clean water to near zero for routine healthcare services. This is because health facilities and personnel have been systematically targeted, thus creating a man-made public health catastrophe which needs immediate international intervention. As a result, the health burdens are rising and there are disease outbreaks. Lack of clean water and sanitation facilities has created a perfect environment for diseases in overcrowded displacements. Immunization programs have been greatly affected and therefore there are gaps in routine vaccinations.

### Rising health burdens and disease outbreaks

The collapse of Gaza’s healthcare infrastructure has contributed to a sharp increase in communicable diseases. Over one million cases of respiratory infections have been recorded since the conflict began, along with increasing cases of diarrhea, acute jaundice syndrome, skin diseases, and acute malnutrition. The lack of clean water and sanitation facilities has created ideal conditions for disease outbreaks, particularly in overcrowded shelters where displaced populations are taking refuge. Immunization programs have been severely disrupted, leading to gaps in routine vaccinations and exposing children to preventable diseases, like polio. Alarmingly, participants reported that malnutrition rates among children under five in northern Gaza have reached 30%, a significant increase from the initial 15% reported earlier in the conflict. The discussions highlighted the pressing need to restore public health surveillance and health information systems, which have collapsed due to the ongoing violence, making it difficult to track and respond to disease outbreaks effectively.

### Critical gaps in emergency and primary healthcare

The discussions underscored gaps in emergency and primary healthcare services in Gaza. Hospitals are operating under extreme conditions with limited fuel and medical supplies, leading to the shutdown of critical services such as intensive care units, operating rooms, and emergency surgical services. Panelists highlighted that healthcare providers are struggling to meet the needs of the injured, pregnant women, children, and the older population, who are among the most vulnerable. The sessions stressed the importance of integrating emergency response planning into primary healthcare services to ensure continuity of care during crises. Furthermore, without adequate fuel supplies, hospitals and clinics will be unable to continue operating, putting thousands of lives at risk.

### Urgent mental health and psychosocial support needs

The need for mental health and psychosocial support was a recurring theme throughout the sessions. The conflict has had a devastating psychological impact on Gaza’s population, particularly children who have witnessed violence and trauma. Furthermore, healthcare workers themselves are facing immense psychological stress, leading to burnout and further affecting their delivery of healthcare services. Panelists emphasized that mental health services must be prioritized alongside physical health services to address the long-term trauma caused by this aggression. Community-based mental health programs were recommended to help build resilience and to support psychosocial well-being. Other recommendations included providing specialized mental health support to healthcare providers (Help the Helpers) to ensure they can continue their vital work in such dire environment.

### Governance and coordination challenges

Governance and coordination challenges in delivering healthcare services were a key focus of the sessions. The ongoing conflict has disrupted local governance structures, making it difficult to coordinate health recovery efforts effectively. The security challenges posed by ongoing military actions from Israel, including attacks on healthcare facilities, the blockade on Gaza’s borders, and restrictions on the movement of people and humanitarian aid, have further hindered the ability to mobilize resources and distribute aid where needed. The panelists also noted that these security barriers severely limit the delivery of essential medical supplies. Strengthening local governance structures was identified as a priority to ensure more effective management and utilization of the scarce healthcare and humanitarian services in Gaza. Participants emphasized the importance of empowering local health authorities to lead the recovery process, ensuring that resources are used efficiently and that healthcare delivery is tailored to the needs of the local population. However, without addressing the security constraints, governance improvements alone will not be sufficient. Ensuring unconditional humanitarian access and protection for health workers and facilities under international humanitarian law is essential to enable effective healthcare recovery and resilience-building.

### Urgent need for resource mobilization and international support

It was also highlighted sustained international support to address the healthcare crisis in Gaza are of high-priority. The discussions emphasized that without immediate and unconditional access to humanitarian aid, including medical supplies and fuel, the healthcare system will continue to deteriorate. The sessions called for the establishment of systematic medical evacuation mechanisms for severely ill patients who need treatment outside of Gaza through secured humanitarian routes. There was also a strong call for international organizations and governments to step up their resource mobilization efforts to ensure both immediate relief and long-term recovery. The importance of a multi-sectoral approach to rebuilding Gaza’s healthcare infrastructure was stressed, where the panelists called for collaboration across health, water, sanitation, and education sectors and across municipalities to address this crisis comprehensively and thoroughly.

### Ensuring health workforce capacity and protection

The health workforce in Gaza has been severely affected by the ongoing conflict, with many healthcare workers displaced or injured, arrested, or killed, further straining the healthcare system. The panelists stressed the loss of numbers of experts and specialized health professionals; and the importance of protecting healthcare workers and facilities in accordance with international humanitarian law. It was highlighted that investing in surge capacity and providing ongoing training programs to equip healthcare workers with the necessary skills to respond to emergencies effectively is very important. Strengthening the health workforce is essential to ensure that Gaza’s healthcare system can withstand future shocks and provide sustainable healthcare services to the population when needed.

### Importance of multi-sectoral collaboration

The discussions emphasized that rebuilding Gaza’s healthcare system requires robust multi-sectoral collaboration. The panelists stressed that addressing the healthcare crisis cannot be done in isolation and must involve coordinated efforts across various sectors, including health, water, sanitation, education, and infrastructure through the strengthening of partnerships between local and international organizations to mobilize resources and expertise. Integrating public health interventions with broader humanitarian responses was also stressed to ensure that the healthcare system recovery is comprehensive and sustainable.

## Discussion

The UN Human Rights Office has documented over 136 attacks on Gaza’s hospitals from October 2023 to June 2024, leading to healthcare system collapse and breaches of international law. The report highlights dire impacts on women and children, with limited prenatal care, which in turn increased maternal and infant mortality. It calls for independent investigations, accountability, and prioritizing healthcare restoration in recovery efforts (6).

The World Health Organization (WHO) reported that attacks on health facilities, which are over 1,000 attacks, have resulted in destruction of hospitals, clinics, and primary healthcare centers and resulted in fatalities and injuries among healthcare workers, further straining service delivery (7). The World Bank and United Nations estimated that, as of January 2024, damage to Gaza’s infrastructure, including healthcare facilities, amounted to approximately $18.5 billion (8). Furthermore, according to the Palestinian MOH, more than 500 medical professionals had been killed during the same period only (9).

With infrastructure collapse, communicable diseases have increased sharply. Over one million cases of respiratory infections have been recorded since the conflict began, along with soaring incidence rates of dysentery, watery diarrhea, and acute respiratory infection (10). Over 33,000 cases of diarrhea have been reported since October 2023, with children under five being disproportionately affected. The lack of clean water and sanitation facilities has created ideal conditions for rapid spread of infectious diseases, particularly in overcrowded shelters where displaced populations are taking refuge. Lack of essential supplies necessary for infection prevention and control along with medications have further compounded the situation. Additionally, the improper management and disposal of medical waste due to disrupted systems have increased the risk of contamination and disease transmission. Immunization programs have been severely disrupted, leading to gaps in routine vaccinations and exposing children to preventable diseases, like polio. Alarmingly, participants at the September 2024 conference reported that malnutrition rates among children under five in northern Gaza had reached 30%, a significant increase from the initial 15% reported earlier in the conflict. The WHO has warned that the combination of hunger and disease could lead to more deaths in Gaza (11).

Hospitals are operating under extreme conditions with limited fuel and medical supplies, leading to the shutdown of critical services such as intensive care units, operating rooms, and emergency surgical services. Several operations were conducted without anesthesia. The UN High Commissioner for Human Rights, Volker Türk, referred to the destruction of hospitals as a ‘human rights catastrophe,’ emphasizing the patterns of targeted attacks on healthcare facilities and the forced removal of patients and staff amidst ongoing hostilities (12). The WHO has warned that hospitals in the Gaza Strip are at a breaking point, with access for emergency medical teams severely hampered by infrastructure damage.

The conflict has had a devastating psychological impact on Gaza’s population, particularly children who have witnessed violence and unimaginable trauma. Mental health providers in Gaza report that the ongoing blockade and violence significantly contribute to psychological distress, particularly affecting children, community well-being, and quality of life (13). Additionally, economic deprivation resulted in limited access to food and water, with limited access to healthcare, and disrupted education further compound this challenge. Strengthening social support networks and investing in community resilience are essential to addressing the long-term mental health impacts in Gaza (5). However, with continuous displacement, community cohesion and resilience became very challenging.

Healthcare workers themselves are facing severe psychological stress, leading to burnout and further affecting their delivery of healthcare services. The United Nations has highlighted that hospitals have become battlegrounds exacerbating the need for mental health and psychosocial support (12).

The ongoing conflict has also disrupted local governance structures, making it difficult to coordinate health recovery efforts effectively. Security challenges, including attacks on healthcare facilities and restrictions on the movement of people and humanitarian aid, have further hindered the ability to mobilize resources and distribute aid where needed. The WHO has called for the protection of humanitarian space in Gaza following serious incidents that have impeded health interventions (14–16).

The healthcare crisis in Gaza necessitates timely and sustained international engagement. Without immediate and unconditional access to humanitarian aid, including medical supplies and fuel, the healthcare system will continue to deteriorate. EMPHNET emphasizes the essential role of fostering partnerships among health authorities, international organizations, and local communities. These collaborative efforts are essential for addressing complex health challenges and building resilient healthcare systems in Gaza (17). Table 1 presents a synthesis of the core public health challenges discussed during the sessions, along with context-specific recommendations derived from expert and frontline insights. Rebuilding Gaza’s healthcare system requires robust multi-sectoral collaboration. Addressing the healthcare crisis cannot be done in isolation and must involve coordinated efforts across various sectors, including health, water, sanitation, education, and infrastructure (18).

**Table 1:** Key Public Health Challenges and Multi-Sectoral Recommendations.

Public health challenge	Recommended action
Destruction of healthcare infrastructure	Immediate ceasefire; restoration of hospitals; reopening of primary health services
Disease outbreaks and disrupted immunization	Re-establish disease surveillance; restore immunization programs; deploy mobile clinics
Mental health crisis and population trauma	Scale up community-based mental health services; support healthcare workers with psychosocial programs
Displacement and overcrowding	Improve WASH services; expand shelter infrastructure; enhance sanitation in temporary housing
Shortages of supplies and health personnel	Facilitate humanitarian corridors; ensure fuel and medicine access; deploy emergency medical teams
Collapse of local governance and coordination	Strengthening local health authorities; improve coordination hubs; ensure protection under international law
Weak international support and aid bottlenecks	Promote multi-sectoral donor coordination; enable rapid resource mobilization and medical evacuations

## Conclusion

The ongoing crisis in Gaza represents a profound public health emergency that demands immediate and sustained attention. In a nutshell, Gaza lacks all the necessities of life, which were systematically destroyed by the war. The precondition for implementing successful and effective responses to preserve life and health is a permanent cease- fire, the withdrawal of the occupation forces, lifting the siege, and allowing freedom of movement and ensuring food security.

The extensive destruction of healthcare infrastructure, compounded by systemic shortages of medical supplies, clean water, and food, has created conditions for the rapid spread of communicable diseases, complications of NCDs as well as a significant deterioration in the nutrition of the population. Although the aggression has pushed Gaza’s fragile healthcare system under extreme strain, it has also demonstrated remarkable resilience. Thus, providing essential medical resources such as medication, medical equipment, and surgical supplies is an immediate priority, while also addressing limitations in governance and coordination mechanisms to ensure effective crisis management and recovery.

The sessions highlighted the urgency of addressing these multifaceted challenges through coordinated, multi-sectoral efforts. Key priorities identified include emergency response planning, primary healthcare, the management of communicable and non- communicable diseases, and the provision of mental health and psychosocial support. The sessions also emphasized the need for resource mobilization, international collaboration, and workforce capacity development to build a resilient healthcare system. This should be implemented, benefiting from the Gaza Strip’s experienced and qualified health human resources. It is imperative to mobilize resources to support them in implementing all medical and public health functions.

The importance of community-based approaches was also highlighted, particularly in mental health interventions and service delivery, as well as in fostering resilience among displaced populations. Additionally, panelists recognized the essential role of empowering local governance structures and ensuring the protection of healthcare workers and facilities in accordance with international humanitarian law. Despite the significant challenges, the sessions reaffirmed the potential for strategic collaboration among international organizations, regional health authorities, and local stakeholders. The adoption of innovative frameworks, such as integrated disease surveillance and the One Health approach, can strengthen preparedness and response capacities. Furthermore, leveraging lessons from global health initiatives and applying them to Gaza’s unique context can facilitate long-term resilience. These factors are urgent in creating a sustainable pathway for recovery.

Ultimately, addressing the healthcare crisis in Gaza requires a focus on immediate humanitarian relief and long-term recovery. This requires bridging significant gaps in healthcare delivery, restoring essential services, and strengthening health governance. By prioritizing equity, fostering partnerships, and committing to sustained investment in Gaza’s healthcare system, the international community has an opportunity to not only alleviate immediate suffering but also lay the foundation for a healthier, more resilient future for Gaza.

## Data Availability

The original contributions presented in the study are included in the article/supplementary material, further inquiries can be directed to the corresponding author/s.
